# Identify of Fast-Growing Related Genes Especially in Height Growth by Combining QTL Analysis and Transcriptome in *Salix matsudana* (Koidz)

**DOI:** 10.3389/fgene.2021.596749

**Published:** 2021-03-31

**Authors:** Guoyuan Liu, Qingshan Yang, Junfeng Gao, Yuwei Wu, Zhicong Feng, Jingke Huang, Hang Zou, Xingzhao Zhu, Yanhong Chen, Chunmei Yu, Bolin Lian, Fei Zhong, Jian Zhang

**Affiliations:** ^1^Key Laboratory of Landscape Plant Genetics and Breeding, School of Life Sciences, Nantong University, Nantong, China; ^2^Shandong Academy of Forestry, Jinan, China

**Keywords:** *Salix matsudana Koidz.*, fast-growing, height growth, RNA-seq, quantitative trait locus

## Abstract

The study on the fast-growing traits of trees, mainly valued by tree height (TH) and diameter at breast height (DBH), is of great significance to promote the development of the forest industry. Quantitative trait locus (QTL) mapping based on high-density genetic maps is an efficient approach to identify genetic regions for fast-growing traits. In our study, a high-density genetic map for the F_1_ population was constructed. The genetic map had a total size of 5,484.07 centimorgan (cM), containing 5,956 single nucleotide polymorphisms (SNPs) based on Specific Length Amplified Fragment sequencing. Six fast-growing related stable QTL were identified on six chromosomes, and five stable QTL were identified by a principal component analysis (PCA). By combining the RNA-seq analysis for the two parents and two progenies with the qRT-PCR analysis, four candidate genes, annotated as *DnaJ, 1-aminocyclopropane-1-carboxylate oxidase 1 (ACO1), Caffeic acid 3-O-methyltransferase 1 (COMT1)*, and *Dirigent protein 6 (DIR6)*, that may regulate height growth were identified. Several lignin biosynthesis-related genes that may take part in height growth were detected. In addition, 21 hotspots in this population were found. The results of this study will provide an important foundation for further studies on the molecular and genetic regulation of TH and DBH.

## Introduction

Forests are the most important source of natural raw material for industries and the environment. The study on the fast-growing traits of trees is of great significance to promote the development of the forest industry. Fast-growing traits, including height growth, diameter growth, volume of wood, growth period, dry weight, and biomass, are complex quantitative traits controlled by multiple genes and environmental factors. High fast-growing trees produce enough raw material to satisfy the industry. Developing high fast-growing cultivars with good wood quality remains a challenge for forest breeding. Till now, a series of studies have focused on genes that play an important role during tree development. However, their roles in genetically controlling fast-growing traits are poorly understood.

Fast-growing traits are both complex and quantitative. They are not only controlled by environmental factors such as light, temperature, water, and fertilizer, but are also controlled by genetic variation, the effect of which is much greater. Among the fast-growing related traits, TH and DBH growth are the most important characteristics of forest trees. Many studies have identified important genes that are involved in the development of TH and DBH. For example, in the stems of woody dicotyledonous plants, the procambium further differentiates outward into phloem cells and inward into xylem cells. In the process of differentiation, the cambium in the bundle between the xylem and phloem still has its meristematic ability (Rose, 2016). About 60 mm from the top of the stem, the stem begins to thicken gradually and the secondary growth of vascular tissue begins (Murmanis, 1970). Studies in poplars have shown that *WUSCHEL*, *CLAVATA*, *SHOOT MERISTEMLESS*, and members of these gene families are involved in cambium primordial cell activity and regulate tree growth (Brand et al., 2000; Gross-Hardt and Laux, 2003; Sarkar et al., 2007). It has also been found that *PXY*, a gene encoding CLV-like LRR-kinase in poplars, plays an important role in maintaining the normal polarity of procambium cells and the structure of the vascular development space (Fisher and Turner, 2007; Fukuda et al., 2007; Hirakawa et al., 2008). In *Arabidopsis thaliana*, it was found that the cambium activity of the mutant hca gene and the secondary growth of the whole plant were hampered, and the expanded secondary growth changed the structure of the stem’s vascular tissue (Pineau et al., 2005). The *Arabidopsis COV1* gene encodes a membrane protein with an unknown function and regulates the proliferation of procambium/cambium. The number of xylems and phloems in the stem of a *COV1* mutant increases along with the number of vascular bundles near the base of the stem (Parker et al., 2003). The *Arabidopsis STM* gene and *BP* gene are the main regulators of stem cell maintenance and cell differentiation in the apical meristem (Groover et al., 2006; Du et al., 2009). The *Arabidopsis HD-ZIP III* gene is involved in regulating the growth of the apical meristem (Floyd et al., 2006) and its homologous gene *PCN* can also slow down the growth of the poplar (Schrader et al., 2004).

Due to its strong tolerance to salt, water, heavy metals, the cold, diseases, and pests, *Salix matsudana* Koidz. is widely distributed around the earth, especially in China. Additionally, the willow has a high biomass, is easy to reproduce, and is rich in variety. It is widely used in artificial forests and its wood is an important raw material in papermaking, gunpowder, construction equipment, particleboard, and other industries. Researchers have already studied gene expression on biomass, salt stress, response to heavy metals, and so on. However, the genetic relationship with fast-growing traits such as TH and DBH is unclear.

In our previous study, an F_1_ population was developed from two *Salix matsudana* Koidz. cultivars with significant differences in TH and DBH (Zhang et al., 2016). The objectives of this study were (1) to locate TH- and DBH-related QTL in the F_1_ population based on its high-density genetic map and a reference genome and (2) to combine the above results with RNA-seq analysis and qRT-PCR analysis to identify candidate genes within stable TH QTL regions.

## Materials and Methods

### Plant Materials and Tissue Collection

Two *Salix matsudana* Koidz. with significantly different TH and DBH were chosen as parents: “9901,” the male parent with the taller TH and bigger DBH and “Yanjiang,” the female with the shorter TH and smaller DBH. The two parents were hybridized to produce F_1_ in 2014. The F_1_ population of 195 plants was grown for DNA extraction at the experimental forest farm in Nantong, Jiangsu, China, in 2015 (Zhang et al., 2016). The branches of 195 F_1_ progenies and the two parents were clipped at 10 cm lengthwise and 1 cm thick and cut into nutrient soil in Nantong University, in March 2020. The clipped branches of a high F_1_ progeny (named as “FH”), a short F_1_ progeny (named as “FS”), and the two parents were cut into nutrient soil in three biological replications for RNA-seq, additionally. The terminals of the stems (0–5 cm) were collected from each replication. The excised stem terminals were immediately frozen in liquid nitrogen and stored at −80°C until use.

### Measurement of Fast-Growing Traits

In November 2018 and 2019, the TH and DBH of the F_1_ population were investigated because the two parents significantly differed in those two traits. In November 2020, only TH of the cuttings of F_1_ population were measured, while little difference was identified on DBH for these cuttings. Additionally, the growing speed of TH (height per year, HPY) and DBH (DBH per year, DPY) were calculated; the phenotypic traits were listed in Supplementary Table S1. The statistical analysis, i.e., the correlation analysis and PCA, were performed by R language.

### RNA Sequencing and Library Construction

Total RNA was extracted from each replication using the Plant RNA Reagent kit (Tiangen, China), according to the manufacturer’s instructions. All RNA samples were quantified using a Nanodrop ND 2000 spectrophotometer (NanoDrop, Thermo, United States). Three RNA samples of each biological replications from stem terminal sample of “9901,” “Yanjian,” “FH”, and “FS” were stored at −80°C. Finally, Illumina sequencing technology (Illumina, San Diego, CA, United States) was employed to perform RNA sequencing by Majorbio (Shanghai, China).

### Analysis of Sequencing Data

The transcriptome reads were processed into clean, full-length reads by removing the low-quality and adapter reads (Chen et al., 2020). The assembled *Salix matsudana* Koidz. (“Yanjiang”) genome sequence was selected as the reference for paired-end reads mapping (Zhang et al., 2020). The clean reads were aligned to genes of the reference genome using the HiSAT2 software^1^ with default parameters (Kim et al., 2019). Then StringTie^2^ was used to detect new transcripts (Pertea et al., 2015). RSEM^3^ was chosen to calculate the fragments per kilobase transcriptome per million mapped reads (FPKM) by normalizing for the length of the gene and for the number of mapped reads. The differentially expressed genes (DEGs) were judged based on the following standards using the Deseq2 package: (1) the FPKM of DEGs should show at least 2-fold changes in expression level between different libraries and (2) the p-adjust value of false discovery rate (FDR) correction with Benjamini–Hochberg should be less than 0.05 (Li et al., 2017; Chen et al., 2020). A BLASTx search was then performed against the NCBI non-redundant protein database to annotate the DEGs. The top three hits (*p* < 0.001) were chosen as the annotation of the potential function of each predicted target. Finally, gene ontology (GO) categories and KEGG pathway analysis were performed to evaluate the potential functions of the targets using Blast2GO (Young et al., 2010). The data that support the findings of this study have been deposited into CNGB Sequence Archive (CNSA) of China National GeneBank DataBase (CNGBdb)^4^ with accession number CNP0001576.

### Linkage Map Construction and Mapping of Fast-Growing Traits

The DNA of 195 F_1_ progeny were extracted, constructed, and sequenced by the Specific Length Amplified Fragment sequencing (SALF-seq) in our previous research. After removing the low-quality reads, the clean reads from each sample were then aligned to the reference genome using Burrows-Wheeler Aligner (BWA) software (set at mem -t 4 -k 32 -M -R) (Li and Durbin, 2009). GATK software was used to call SNPs for all of the samples (McKenna et al., 2010). SNP markers with segregation patterns of ab × cd, ef × eg, hk × hk, nn × np, lm × ll in the parents were used to construct a linkage map. SNP markers with no more than 15% missing data in the F_1_ population and a *p*-value of segregation distortion of less than 0.05 were selected to construct a linkage map (Liu et al., 2019). The SNP markers were first divided into 38 groups according to the position mapped on the 38 chromosomes of the reference genome of “Yanjiang.” JoinMap 4.0 was used for the linkage map construction (van Ooijen, 2006). Interval mapping (IM) method was employed to detect TH-, DBH-, and PCA-related QTL using MapQTL 6 (Bokore et al., 2019). The parameters were set to 1 cM of the step and 1,000 permutations were taken as the LOD threshold. QTL were named according to McCouch et al. (1997). MareyMap was applied to construct a recombination map, which displayed a smooth curve with the Loess method (Rezvoy et al., 2007). Regions no less than 50 cM/Mb were regarded as recombination hotspots (Liu et al., 2019).

### Identification of Candidate Genes and Expression Pattern Analysis

The DEGs and TH QTL were co-localized onto the reference genome based on a BLAST search. Total RNA of stem terminals from “9901,” “Yanjian,” “FH,” and “FS” were extracted. The One-Step SYBR Primer Script Plus RT-PCR kit (Takara, Beijing, China) was used according to the manufacturer’s instructions to conduct a qRT-PCR analysis of the candidate genes. The *Actin* gene was used as an internal control (Chen et al., 2020). All primers are listed in Supplementary Table S3.

## Results

### Determination of Fast-Growing Traits in the F1 Population

**FIGURE 1:**
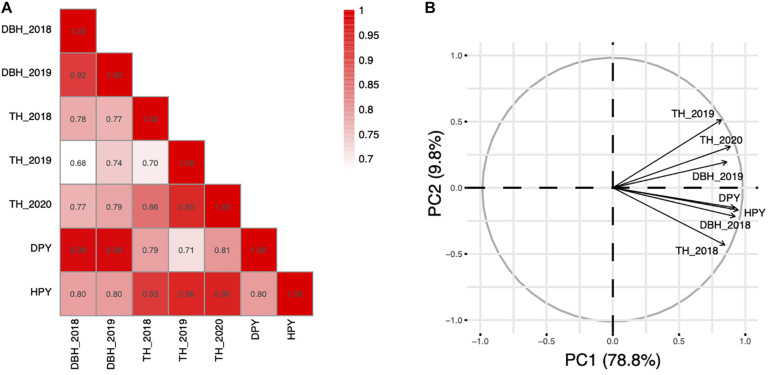
Correlation and PCA analysis among the fast-growing related traits. **(A)** Correlation analysis among the fast-growing related traits. **(B)** PCA analysis of the fast-growing related traits.

The traits of the F_1_ population are summarized in Table 1. As shown, there is a significant difference between the TH and DBH of the two parents. Both TH and DBH exhibited transgressive segregation in the segregating population. The heritability of HPY and DPY were 0.877 and 0.853, respectively. For the lack of replicates in each environment, the heritability of TH and DBH were not performed. Furthermore, TH, DBH, HPY, and DPY were significantly correlated (Figure 1A), indicating possible pleiotropic effects of the same QTL for these fast-growing traits. According to the PCA, which was performed to detect the common factors underlying trait variation, all traits showed high positive loadings on PCA1, which can explain 78.8% of the variance of traits (Figure 1B). This result suggests that F_1_ plants with high PCA1 scores in this population exhibited tall TH and high DBH. This corresponds to a trade-off relationship between TH and DBH. The PCA2 only explained a 9.8% variance. The loading on different environments was different, suggesting that PCA2 is representative of a different environment. Additionally, the result also showed that TH and DBH were stable in different years, which is consistent with the correlation analysis.

**TABLE 1:** Performance and analysis of fast-growing traits in two parents and F_1_ population.

Trait	Environment	Parents			F_1_ population			
		Male	Female	Significant	Range	Means	Kurtosis	Skewness

		Parent (9901)	Parent (Yanjiang)					
DBH(cm)	2018	3.66	3.40	*	0.60−8.20	3.59	0.20	0.68
	2019	5.49	4.99	*	3.20−13.10	5.11	0.53	0.78
Height(cm)	2018	584.72	442.23	**	134.00−663.00	498.59	0.68	-1.11
	2019	768.47	600.94	**	198.11−1208.45	695.51	-0.13	-0.07
	2020	249.94	145.37	**	90.00−290.00	190.42	-0.13	0.07
DPY(cm)	Combine	1.01	0.92	*	0.15−2.34	0.93	0.27	0.68
HPY(cm)	Combine	103.62	81.27	**	33.50−199.97	128.66	0.08	-0.64

Significant difference at p < 0.01 and p < 0.05 were marked as ** and *, respectively.

### High-Throughput Linkage Map Construction

A high-density linkage map containing a total of 5,956 SNPs divided into 38 linkage groups (LGs) was constructed. The total genetic distance was 5,484.07 cM and the average distance between adjacent markers was 0.92 cM (Table 2). The genetic distances of the 38 LGs ranged from to 96.02 cM (B09) to 252.34 cM (A16). The SNP markers mapped to each LG varied from 72 (A19) to 332 (A16). Among the 38 LGs, the genetic distance of chromosome A16 and D16 were longer than 200 cM and only A17 and A19 contained no more than 100 SNP markers. Chromosome B10 contained the largest gap (19.89 cM). Overall, the markers were randomly distributed on 38 chromosomes, which means the linkage map is suitable to perform further QTL analysis on the whole genome.

**TABLE 2:** Detailed information on the high-density genetic map.

Chromosome	Length (cM)	Number of mapped markers	Average interval (cM)	Largest gap (cM)	Recombination rate (cM/Mb)	Collinearity ratio
A01	156.42	103	1.52	8.58	13.45	99.02%
A02	174.32	218	0.80	13.78	10.35	98.36%
A03	137.38	164	0.84	5.29	11.04	99.56%
A04	153.93	142	1.08	6.80	10.37	98.35%
A05	151.98	197	0.77	7.76	10.53	96.97%
A06	182.24	223	0.82	10.83	8.45	98.48%
A07	126.16	152	0.83	8.59	8.86	97.37%
A08	142.91	162	0.88	4.74	11.59	98.64%
A09	103.07	110	0.94	7.62	9.31	97.65%
A10	155.46	151	1.03	6.73	9.37	98.47%
A11	121.46	100	1.21	13.03	10.53	97.36%
A12	129.50	114	1.14	6.16	11.78	95.19%
A13	138.26	171	0.81	6.23	11.12	97.37%
A14	121.48	120	1.01	5.14	13.08	94.85%
A15	111.51	120	0.93	6.82	9.93	94.14%
A16	252.34	332	0.76	7.30	8.88	98.67%
A17	110.12	79	1.39	11.64	12.06	97.34%
A18	113.58	104	1.09	11.38	9.42	89.44%
A19	104.84	72	1.46	9.09	11.66	95.86%
At sub-genome	2686.95	2834	0.95	13.78	10.33	97.01%
B01	165.44	201	0.82	8.02	8.94	98.98%
B02	175.73	193	0.91	6.81	9.76	97.40%
B03	144.46	135	1.07	9.72	10.79	95.20%
B04	166.48	232	0.72	5.23	9.74	98.11%
B05	175.40	294	0.60	10.47	9.07	97.32%
B06	174.28	257	0.68	6.8	8.01	98.04%
B07	137.23	136	1.01	16.77	11.94	97.72%
B08	144.56	135	1.07	7.55	11.91	98.48%
B09	96.02	113	0.85	5.94	8.65	97.65%
B10	158.97	135	1.18	19.89	13.56	97.80%
B11	129.25	109	1.19	7.67	11.08	96.89%
B12	106.10	125	0.85	7.48	8.72	94.36%
B13	150.69	188	0.80	8.33	10.71	96.60%
B14	129.30	105	1.23	7.38	14.69	93.98%
B15	148.81	118	1.26	9.95	15.41	92.36%
B16	240.00	278	0.86	8.52	8.60	99.28%
B17	123.62	152	0.81	6.39	9.26	97.19%
B18	123.86	112	1.11	12.62	11.52	97.17%
B19	106.93	104	1.03	10.54	10.82	94.36%
Bt sub-genome	2797.12	3122	0.90	19.89	10.25	96.78%
Total	5484.07	5956	0.92	19.89	10.29	94.70%

**FIGURE 2:**
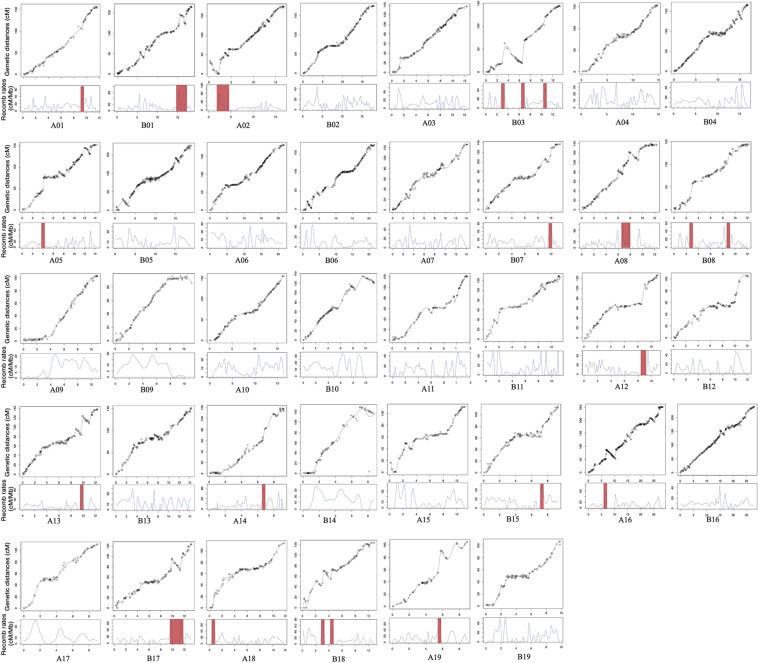
Correlation among genetic and physical maps, estimated local recombination rates, and their distribution in chromosomal rearrangement regions. The red shadow represents recombination hotspots.

Collinearity was measured to assess the quality of this genetic map (Figure 2 and Table 2). The results indicated that most LGs in this newly constructed linkage map have high collinearity with the physical map of the reference genome “Yanjiang.”

We also measured the distribution of genome-wide variation of recombination rates. As shown in Figure 2, the recombination rate was found to vary across chromosomes. Twenty-one recombination spikes were found in the centromeric regions of A01, A02, A05, A08, A12, A13, A14, A16, A18, A19, B01, B03, B07, B08, B15, B17, and B18. The average recombination rate for each chromosome also showed significant differences, ranging from 8.01 to 15.41 cM/Mb (Table 2), with an overall genome-wide recombination rate of 10.29 cM/Mb. On average, the recombination rate was similar between the At and Bt sub-genomes. Most recombination spikes were induced by chromosome segment inversions, such as A01, A02, A08, A13, A16, B01, B03, B07, B17, B18, and B19. Several recombination hotspots were detected in both homoeologous chromosomes, i.e., A01 and B01 and A08 and B08. Except for the segment inversion, the average genome-wide recombination rate was not random, as the distal chromosomal regions showed higher recombination rates than the proximal regions in most chromosomes.

### QTL Mapping of Fast-Growing Traits in the F1 Population

**FIGURE 3:**
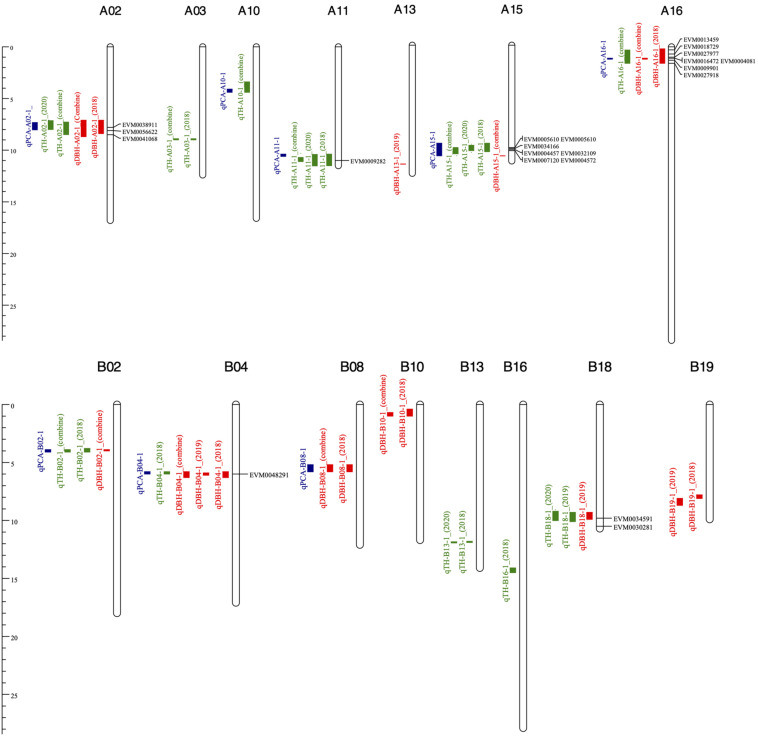
QTL of TH, DBH, and PCA on the physical map. TH, DBH, and PCA QTL are marked in green, red and blue, respectively.

Based on the high-density genetic map and the PCA, a total of 29 fast-growing and PC1-related QTL were identified on 15 chromosomes: A02, A03, A10, A11, A13, A15, A16, B02, B04, B08, B10, B13, B16, B18, and B19 (Figure 3). These 29 QTL contained 10 DBH QTL, 11 TH QTL, and 8 PCA QTL, each explaining 5.8–10% of the phenotypic variation (PV). Among the 29 QTL, six and seven stable QTL related to DBH and TH in at least two environments, respectively, were detected (Table 3). Moreover, seven regions of the genome contained both TH QTL and DBH QTL, which indicates that these six multi-effect regions could affect both TH and DBH. According to the distribution of these 29 QTLs, chromosomes A02, A15, A16, B02, B04, and B18 contained both TH- and DBH-related genes. However, we also detected QTLs on chromosomes A03, A10, A11, B13, and B16 only related with TH, and QTLs on chromosomes A13, B08, B10, and B19 that only regulate DBH. These chromosomes were detected related with TH or DBH, respectively. The result may provide scientific basis for genetic engineering and molecular marker-assistant breeding of TH and DBH. The eight PC1-related QTL were detected on eight chromosomes. We then compared whether these eight regions overlapped with the seven multi-effect regions. As a result, five of the eight (62.50%) QTL were located in the same region as the seven multi-effect regions on the reference genome, and all five QTL could be detected in the two environments. The result indicated that the PCA is powerful enough to detect multi-effect QTL for high correlated traits. Based on the PCA, five stable QTL that could affect both DBH and TH were detected, which may explain the significant positive relationship between TH and DBH. The five stable QTL were located on chromosomes A02, B02, B04, A15, and A16 (Figure 3).

**TABLE 3:** Detailed information of 29 QTL for fast-growing in F_1_ population.

Traits	QTL	Environment	Chromosome	LOD	*R*^2^ (%)	95% confidence interval (cM)	Physical interval (Mb)
DBH	qDBH-A02-1	2018	A2	3.62	9	67.84−84.53	7.11−8.41
		Combine	A2	3.48	8.87	67.84−85.09	7.11−8.69
	qDBH-B02-1	Combine	B2	2.5	5.8	55.40−55.40	3.86−4.00
	qDBH-B04-1	2018	B4	3.37	7.7	61.58−73.34	5.80−6.32
		2019	B4	2.75	7.3	65.58−69.48	5.85−6.05
		Combine	B4	3.97	9.3	60.58−73.34	5.78−6.32
	qDBH-B08-1	2018	B8	3.67	8.6	72.78−75.78	5.24−5.76
		Combine	B8	3.31	7.8	73.78−75.28	5.24−5.76
	qDBH-B10-1	2018	B10	2.94	6.8	6.53−11.10	0.39−0.96
		Combine	B10	2.74	6.3	7.53−10.10	0.74−0.96
	qDBH-A13-1	2019	A13	2.73	8.5	126.54−127.54	11.45−11.50
	qDBH-A15-1	Combine	A15	2.73	6.3	108.92−110.42	10.68−10.70
	qDBH-A16-1	2018	A16	2.73	7.7	0−8.91	0.15−1.57
		Combine	A16	2.5	5.9	0.15−1.57	1.10−1.12
	qDBH-B18-1	2019	B18	2.78	7.8	118.00−120.45	9.29−9.91
	qDBH-B19-1	2018	B19	2.58	5.9	71.05−78.29	7.83−8.12
		2019	B19	2.72	6.9	78.06−87.63	8.12−8.72
PCA	qPCA-A01-1	PCA	A2	3.02	7.2	68.00−80.13	7.11−7.79
	qPCA-B02-1	PCA	B2	2.51	5.8	55.37−55.40	3.86−4.06
	qPCA-B04-1	PCA	B4	3.24	7.7	62.58−72.71	5.82−6.02
	qPCA-B08-1	PCA	B8	2.92	6.9	73.78−75.28	5.24−5.76
	qPCA-A10-1	PCA	A10	2.52	6.9	38.99−42.52	4.10−4.44
	qPCA-A11-1	PCA	A11	2.94	7.3	97.62−103.25	10.35−10.55
	qPCA-A15-1	PCA	A15	2.73	6.9	89.97−109.42	9.46−10.70
	qPCA-A16-1	PCA	A16	2.51	5.9	0.15−1.57	1.10−1.12
TH	qTH-A02-1	2020	A2	4.06	10	66.46−77.58	7.09−7.67
		Combine	A2	2.65	6.2	71.97−74.28	7.26−7.46
	qTH-B02-1	2018	B2	2.61	6	53.82−55.88	3.80−4.06
		Combine	B2	2.57	5.9	55.37−55.40	3.86−4.06
	qTH-A03-1	2018	A3	2.66	6.6	102.79−105.15	8.91−9.03
		Combine	A3	2.67	6.7	102.79−105.15	8.91−9.03
	qTH-B04-1	2018	B4	3.02	7.9	63.58−72.71	5.83−6.02
	qTH-A10-1	Combine	A10	3.13	7.7	33.37−42.52	3.42−4.44
	qTH-A11-1	2018	A11	2.82	7	98.07−119.51	10.36−11.50
		2020	A11	3.37	8.4	98.27−105.57	10.36−11.04
		Combine	A11	3.23	7.9	95.62−104.90	10.34−10.70
	qTH-B13-1	2018	B13	2.66	6.2	106.14−114.08	10.53−10.82
		2020	B13	3.08	7.7	105.34−117.11	10.53−10.82
	qTH-A15-1	2018	A15	3.01	7.7	89.97−104.17	9.46−10.32
		2020	A15	3.54	8.8	95.71−103.07	9.79−10.27
		Combine	A15	2.9	6.7	106.14−109.42	10.53−10.70
	qTH-A16-1	Combine	A16	2.59	6.2	0.15−1.57	1.10−1.57
	qTH-B16-1	2018	B16	2.83	6.5	124.90−128.80	14.11−14.45
	qTH-B18-1	2019	B18	2.85	7.4	118.00−121.26	9.29−10.06
		2020	B18	3.82	9.5	112.78−120.12	8.98−9.91

Fifteen of the 29 QTL were located on the At sub-genome and the remaining 14 QTL were located on the Bt sub-genome. Both TH QTL and DBH QTL were detected on chromosomes A02, B02, B04, A15, A16, and B18. Interestingly, for the other nine chromosomes, the At sub-genome chromosomes (A03, A10, and A11) contained more TH QTL, while most DBH QTL were located on the Bt sub-genome chromosomes (B08, B10, and B19). TH QTL on A03, A10, A11, and A15 could not be identified on its homoeologous chromosomes in the Bt sub-genome. Similarly, chromosomes A08, A10, and A19 also did not contain DBH QTL. By comparing the QTL distribution on the two sub-genomes, we could draw a conclusion that for this population, one-third of the QTL region could affect both TH and DBH and for the single effect QTL, the At sub-genome contains more TH-related QTL, while the Bt Sub-genome contains more DBH-related QTL.

### Comparative Transcriptome Analysis of TH

**FIGURE 4:**
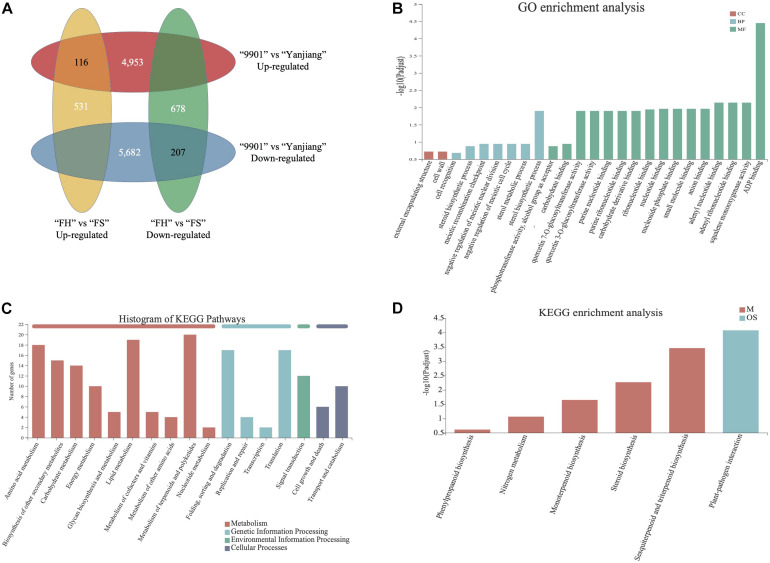
GO and KEGG analysis of differentially expressed genes (DEGs). **(A)** Venn analysis of DEGs. **(B)** GO enrichment analysis of 323 DEGs. **(C)** Statistics analysis of KEGG pathway among the 323 DEGs. **(D)** KEGG enrichment analysis of 323 DEGs.

We performed a transcriptome analysis to understand the molecular mechanism of TH. By sequencing the stem terminal of “FH,” “FS,” “9901,” and “Yanjiang,” a total of 39,327 genes were detected to be expressed in at least one library. This result suggests that 67.99% of the predicted genes (a total of 57,841 gene models in “Yanjiang”) are expressed in the stem terminals. After trimming off the adapter sequences and removing the low-quality reads, we obtained 42,080,544–58,249,766 clean reads for the 12 libraries, with a single read length of 90 bp and Q20 and Q30 percentage (percentage of sequences with sequencing error rates lower than 1 and 0.1%) over 97 and 93%, respectively (Supplementary Table S2). The DEGs in both parents and the two progenies were then identified using the threshold FDR ≤ 0.05 and the absolute value of log2-fold change ≥1 (Li et al., 2017; Chen et al., 2020). As a result, a total of 10,635 and 1,209 DEGs were identified between two parents and two progenies, respectively. Among the 10,635 DEGs, 4,953 genes expressed higher in “9901” than “Yanjiang” and 5,682 genes expressed higher in “Yanjiang” than “9901.” For the two progenies, 531 genes expressed higher in “FH” than “FS” and 678 genes expressed higher in “FS” than “FH”. According to the Venn analysis, 116 DEGs showed high expression levels in both “9901” and “FH” and another 207 DEGs expressed higher in “Yanjiang” and “FS” (Figure 4A). Since most DEGs expressed higher in low fast-growing cultivar, we may infer that most DEGs have a negative relationship with fast-growing traits.

The 323 DEGs (116 up-regulated and 207 down-regulated) in “9901” and “FH” then underwent the GO and KEGG enrichment analysis. As shown in Figure 4B, most DEGs were enriched in the molecular function term. ADP binding was the most enriched, followed by adenyl nucleotide binding, squalene monooxygenase activity, and adenyl ribonucleotide binding, among others. For the biological process, the sterol biosynthetic process was the most enriched. A cell wall and an external encapsulating structure were detected in the cellular components. For the KEGG enrichment analysis, most identified pathways were enriched in metabolism (Figure 4C). Only sesquiterpenoid and triterpenoid biosynthesis, steroid biosynthesis, monoterpenoid biosynthesis, nitrogen metabolism, and phenylpropanoid biosynthesis pathways were identified with significant enrichment levels (Figure 4D). The results of the GO and KEGG analysis indicate that terpenoid-, steroid- and phenylpropanoid-related pathways may play important roles in TH growth.

### Identification of Candidate Genes for TH

To further understand the genetic mechanism of *Salix matsudana* Koidz. on fast-growing traits, we co-located the DEGs and 11 TH QTL on the reference genome. Our hypothesis is that only these differentially expressed genes that are located in QTL regions may be genetically associated with TH. Based on the reference genome, 228 of the 323 DEGs were mapped on 38 chromosomes and 95 of the 323 DEGs were mapped on scaffolds. Of the 228 DEGs, 18 could be co-located with seven stable TH QTL on chromosomes A02, A11, A15, A16, B04, and B18. The 18 co-located DEGs were annotated as plastid-lipid-associated proteins: *DnaJ*, Berberine bridge enzyme, *ACO1*, *COMT1*, and NAC and WAKY transcription factors, among others (Supplementary Table S4). Most (14) of these 18 genes showed higher expression levels in “Yanjiang” and “FS” than “9901” and “FH,” which means that these genes have a negative relationship with TH. For example, *ACO1, COMT1, DnaJ*, and several transcription factors showed a negative relationship with TH. The remaining four genes showed a positive relationship with TH, including genes encoding DIR6 and plastid-lipid-associated protein, among others.

**FIGURE 5:**
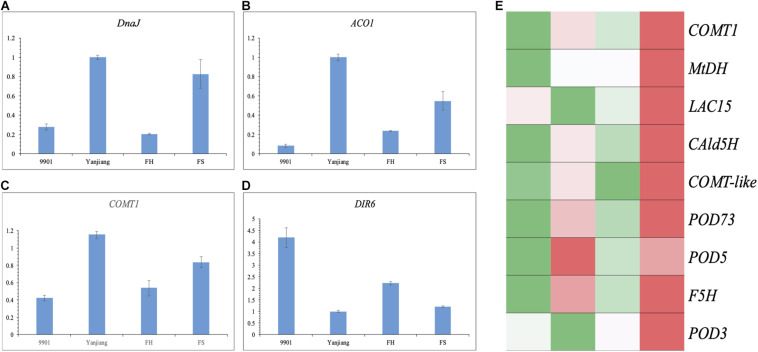
The expression trend of candidate genes and lignin biosynthesis related genes in the two parents and two progenies. **(A–D)** qRT–PCR analysis of the transcript levels of candidate genes in the two parents and two progenies. The Y-axis represents the relative expression level, and the X-axis represents different cultivars. The error bars denote the standard error (SE). **(E)** Heatmap analysis of lignin biosynthesis related genes in the two parents and two progenies.

According to the annotations, four genes were selected as possible candidate genes to perform a qRT-PCR analysis. The *DnaJ* gene for *qTH-A15-1*, the *ACO1* gene for *qTH-A11-1*, and the *COMT1* gene for *qTH-A16-1* showed higher levels of expression in the stem terminals of “Yanjiang” and “FS” than those of “9901” and “FH”. The result indicates that the expression of these genes may suppress the TH growth (Figures 5A–C). Another gene encoding *DIR6* for *qTH-A02-1* was expressed at a higher level in “9901” and “FH” than in “Yanjiang” and “FS” (Figure 5D), displaying a positive relationship with TH. Interestingly, both *DIR6* and *COMT1* were associated with phenylpropanoid or lignin biosynthesis. However, these two genes showed an opposite expression trend. Another candidate gene, *ACO1*, has already been verified to regulate the biosynthesis of ethylene in *Arabidopsis, Popolus*, and *Solanum*, among other plants. Previous studies also found that ethylene could affect the biosynthesis of lignin through activating the downstream transcription factor (EIN and ERF/AP2, among others) or other proteins. We then verified the genes on the pathway of lignin biosynthesis from the 323 DEGs, according to the annotation. As a result, nine genes were identified, annotated as mannitol dehydrogenase (MtDH), ferulic acid 5-hydroxylase (F5H), coniferyl aldehyde 5-hydroxylase 2 (CAld5H), laccase (LAC), and peroxidase (POD), among others. Most of them were expressed higher in “Yanjiang” and “FS” than in “9901” and “FH” (Figure 5E), which is consistent with the expression of *ACO1* and *COMT1*. Lignin is known to take part in cell wall formation, and so the regulation of lignin biosynthesis may affect the development of cells and bring the stage of secondary growth forward.

## Discussion

### TH and DBH in *Salix matsudana* Koidz

*Salix* is known for its versatile use in industries (papermaking, gunpowder, and particleboard, among others) and for ecological purposes, such as afforestation in the city and coastal beach-lands (Zhang et al., 2017). The physiological and biochemical traits on *Salix*, such as nitrogen economy, leaf senescence, bud burst, enzymolysis saccharify, salicin, and insect resistance, have already been studied (Sulima et al., 2009; Brereton et al., 2010; Höglund et al., 2012; Berlin et al., 2014; Ghelardini et al., 2014). Both *Salix* and *Populus* belong to the family of *Salicaceae*. Many researchers have already studied the wood growth of *Populus* (Dubois et al., 2018). However, there remains a lack of information on the wood growth of *Salix matsudana* Koidz. *Salix matsudana* Koidz. is a tetraploid forest tree and has a much more complex genetic mechanism than other diploid forest trees. It is also an ideal model system for studying plant polyploidization (Zhang et al., 2020). In this study, TH and DBH were measured in the F_1_ population during the fourth and fifth year after seeding and 8-month- old cuttings. However, few differences were identified on DBH for these cuttings. To avoid the environmental effect, HPY and DPY were also measured and showed a significant correlation with TH and DBH. While calculating the heritability, for the lack of replicates in each environment, only the heritability of HPY and DPY were measured. Both traits showed high heritability. It is understandable that a bigger DBH could provide stronger mechanical support for trees and result in a taller TH. However, it was found that DBH and TH are determined by different growth patterns. DBH is mainly determined by secondary growth, which includes secondary xylem and phloem thickening, cell anticlinal division, and cell wall thickening (Chaffey et al., 2002; Helariutta and Bhalerao, 2003). TH is mainly determined by stem apical meristem (SAM) cell growth and division in primary growth. Stem cells in the central region of SAM produce various types of vascular cells through continuous division, which in turn promote primary growth (Altamura et al., 2001; Little et al., 2002; Ye et al., 2002). The relationship between TH and DBH is also determined by primary growth and secondary growth. To further understand the relationship between TH and DBH and discover the genetic mechanism difference on TH and DBH, we first analyzed the genetic mechanism on TH and DBH by combining the PCA. A total of seven QTL regions that could affect both TH and DBH were determined, which could explain the high positive correlation between TH and DBH. The QTL mapping result of the PCA is highly consistent with the multi-effect QTL, which indicates that the PCA is able to understand positively correlated traits (Yano et al., 2019). However, there still remains the question of whether multi-effect genes are located in these seven QTL regions or TH-related genes and DBH-related genes are located closely on the genome. To solve this question, more experimental data and a finer mapping of TH and DBH are needed.

### QTL of Fast-Growing and Recombination Hotspots in *Salix matsudana* Koidz

Based on the reference genome of “Yanjiang,” we re-analyzed the genetic map of the F_1_ population. Only the SNPs that could be mapped onto the chromosomes of the genome were selected to construct the genetic map. According to the phenotypes of fast-growing traits, we identified 21 QTL, including 10 DBH QTL and 11 TH QTL. For these QTLs, the PV were ranged from 5.8 to 10%, which is not treated as major QTL in most crops. It might be because the population of *Salix matsudana* Koidz is an F_1_ population, which has a lower additive genetic variance than F_2_ or RIL populations. The PV of this population is similar to *Populus* F_1_ populations previously reported (Mousavi et al., 2016; Liu et al., 2017).

Additionally, six multi-effect QTL regions were identified; the At and Bt sub-genomes contained the same numbers of multi-effect QTL regions. Except for these six multi-effect QTL, three DBH QTL were located on the Bt sub-genome, while only one DBH QTL was located on the At sub-genome. For TH, the At and Bt sub-genomes contained three and two QTL, respectively. We also compared whether QTL is located on homoeologous chromosomes. As a result, only A02 and B02, A10 and B10, A13 and B13, and A16 and B16 showed partial consistency on QTL distribution. While both sub-genomes contained several stable QTL on TH and DBH, the At sub-genome contained more TH QTL and more DBH QTL were detected on the Bt sub-genome. The results indicated that some DBH QTL on the At sub-genome and some TH QTL on the Bt sub-genome were lost under the evolution and selection. We first compared the difference in QTL distribution between the two sub-genomes in *Salix matsudana* Koidz. Although more genetic and natural populations are needed to confirm the differentiation of the sub-genomes, this study proved that the At and Bt genomes were different under artificial selection and natural selection.

Based on the high-density genetic map and the reference genome, we identified 21 recombination hotspots on 17 chromosomes. Previous studies have reported that recombination in *Arabidopsis* (Osman et al., 2011), maize (Schnable et al., 2009), and *Triticum aestivum* (Arbeithuber et al., 2015) usually occurs in intergenic regions and around transposons. This study identified the genome-wide recombination rates, which is helpful to understand the genetic variation. Additionally, lots of segment insertions were detected to be distributed on most chromosomes in this population. The most likely reason for this is the genotypic difference between the two parents.

### A Potential Functional Network of Genes Associated With TH

According to the transcriptomes, 10,635 and 1,209 DEGs were identified between the two parents and two progenies. “Yanjiang” and “9901” were different varieties while “FH” and “FS” contained similar genetic backgrounds. By overlapping the two DEGs, we narrowed the DEGs into 323 genes. The present study identified four DEGs, co-localized with TH QTL, that may be associated with TH growth. The *ACO1* gene located in *qTH-A11-1* was annotated to directly regulate the biosynthesis of ethylene. Several studies have already reported that ethylene could affect cell expansion and the activity of cambium (Dubois et al., 2018). Many ethylene biosynthesis-related enzymes and transcription factors in *Populus* have been proven to express wood tissue (Seyfferth et al., 2018). Other studies have shown that ethylene participates in cell growth and differentiation in cambium and affects the secondary growth of stems (Pesquet and Tuominen, 2011; Felten et al., 2018; Harkey et al., 2019). The content of ethylene could regulate the cell division in meristems and affect the formation of tension wood in *Populus* (Andersson-Gunneras et al., 2003; Love et al., 2009). The up-regulation of the *ACO1* gene could promote ethylene production and induce cotton fiber cell elongation (Shi et al., 2006). According to the expression pattern of *ACO1* in the two parents and two progenies, the high expression level of *ACO1* may reduce the TH growth of *Salix matsudana* Koidz. by regulating the ethylene biosynthesis. A gene encoding the DnaJ protein was detected to be differentially expressed in *qTH-A15-1*. Previously, *DnaJ* was mostly identified as a response to heat stress or other environmental stimulation (Orme et al., 2001; Walsh et al., 2004; Liberek et al., 2008). Our results showed that the *DnaJ* gene may negatively regulate TH growth, due to the high expression of this gene in “Yanjiang” and “FS”. In yeast, the DnaJ protein was reported to regulate the formation of tubulin (Oka et al., 1998), which could determine the direction of cell expansion. Interestingly, 17 of the 18 candidate genes were located in multi-effect QTLs, indicating that these genes might also regulate DBH. For most of the single TH-related chromosomes, we did not detect many DEGs. This may be because the genetic background of “FH” and “FS” were similar and other loci on chromosomes, such as miRNAs and long non-coding RNAs, may also regulate TH. We also identified several lignin-related genes that were differentially expressed. Moreover, two of them could co-localize in stable TH QTL, as seen in *COMT1* in *qTH-A16-1* and *DIR6* in *qTH-A02-1. COMT1* was annotated as a catalyst phenylalanine for coniferyl alcohol (CA) in a phenylalanine pathway, which was the first step to lignin biosynthesis. DIR could then catalyze CA into secoisolariciresinol (SECO) rather than normal lignans (Boerjan et al., 2003; Mazzaferro et al., 2015; Xiao et al., 2015). Our results showed that the down-regulation of *COMT1* and up-regulation of *DIR6* could result in a low expression level of normal lignin. Our study suggests that the reduction of lignin biosynthesis might promote TH growth. We then measured the expression level of other phenylalanine pathway genes. Most lignin biosynthesis genes (*Mannitol dehydrogenase, F5H, peroxidase, etc.*) slowed a low expression level in “9901” and “FH”, which is consistent with the hypothesis. Although further experiments, such as overexpression or CRISPR-Cas9 silencing, are needed to confirm roles in TH, the present QTL analysis, comparative transcriptome analysis, and qRT-PCR analysis showed that these several lignig-related genes may be important for TH and DBH.

## Conclusion

In our study, two varieties were chosen as two parents to construct F_1_ populations to detect fast-growing related (TH and DBH) QTL and genes. Based on the high-density genetic map, five DBH- and eight TH-related stable QTL were identified. A PCA was undertaken to confirm the multi-function stable QTL. The further use of our previous RNA-seq data for the two parents and two F_1_ progenies, followed by the qRT-PCR analysis, detected four candidate genes that may regulate TH. Several lignin biosynthesis-related genes were found to be differentially expressed. Furthermore, 21 recombination hotspots were identified in our population. These results provide an important foundation for further studies on the molecular and genetic regulation of the fast-growing traits of forest trees.

## Data Availability Statement

The datasets presented in this study can be found in online repositories. The names of the repository/repositories and accession number(s) can be found below: “CNGB Sequence Archive (CNSA) of China National GeneBank DataBase (CNGBdb) (https://db.cngb.org/cnsa/) with accession number CNP0001576.”

## Author Contributions

JZ and GL conceived and designed the experiments. GL, JG, YW, ZF, JH, HZ, and XZ performed the experiments. GL, YC, CY, BL, and FZ analyzed the data. GL, QY, and JZ wrote the manuscript. All authors contributed to the article and approved the submitted version.

## Conflict of Interest

The authors declare that the research was conducted in the absence of any commercial or financial relationships that could be construed as a potential conflict of interest.

## Footnotes

1 https://daehwankimlab.github.io/hisat2/

2 http://ccb.jhu.edu/software/stringtie/

3 http://deweylab.github.io/RSEM/

4 https://db.cngb.org/cnsa/
